# Geriatric Psychiatry

**DOI:** 10.1192/pb.bp.113.043851

**Published:** 2014-06

**Authors:** Kathryn Milward

Miller & Solai’s *Geriatric Psychiatry* is part of the Pittsburgh Pocket Psychiatry series. It has the look and feel of an Oxford medical handbook - its formatting and font is identical. However, the *Oxford Specialist Handbooks in Psychiatry: Old Age Psychiatry*^1^ pre-dates this text by 4 years. This raises the question of which is a better buy?

The major difference between the two books is that *Geriatric Psychiatry* is an American text and thus references US guidelines. Consequently, DSM is used throughout, replacing ICD criteria.

This is an ambitious book aspiring to cover the breadth of geriatric psychiatry in 16 chapters. It covers core topic areas but also dedicates chapters to additional aspects of old age psychiatry, for example the psychiatric management of chronic pain and substance use disorders. The detail dedicated to subject areas is inconsistent, with greater depth reserved for more esoteric topics, often at the expense of core subjects. One notable example is the ‘brief behavioural treatment for insomnia’ which was developed by the author of the sleep disorder chapter. More space is dedicated to this treatment than to psychosis in Alzheimer’s disease, mood disorder, delirium and schizophrenia combined. Although some chapters are very brief, each includes a comprehensive further reading list and multiple-choice questions.

Several chapters are succinct, well written and relevant. The chapter on pharmacotherapy principles in ageing is particularly effective, utilising concise tables and clear key-fact boxes. The final chapter on psychosocial factors of health and quality of life is thought-provoking and interesting.

Ultimately, this is a handbook designed for an American audience. Personally, as a UK trainee I would choose to purchase the *Oxford Specialist Handbooks in Psychiatry: Old Age Psychiatry*, but *Geriatric Psychiatry* may be a useful adjunct.
